# Phytohormones and Beneficial Microbes: Essential Components for Plants to Balance Stress and Fitness

**DOI:** 10.3389/fmicb.2017.02104

**Published:** 2017-10-31

**Authors:** Dilfuza Egamberdieva, Stephan J. Wirth, Abdulaziz A. Alqarawi, Elsayed F. Abd_Allah, Abeer Hashem

**Affiliations:** ^1^Leibniz Centre for Agricultural Landscape Research, Institute of Landscape Biogeochemistry, Müncheberg, Germany; ^2^Plant Production Department, College of Food and Agricultural Sciences, King Saud University, Riyadh, Saudi Arabia; ^3^Botany and Microbiology Department, College of Science, King Saud University, Riyadh, Saudi Arabia; ^4^Mycology and Plant Disease Survey Department, Plant Pathology Research Institute, Giza, Egypt

**Keywords:** abiotic stress, plant microbiome, metabolites, phytohormones

## Abstract

Plants are subjected to various abiotic stresses, such as drought, extreme temperature, salinity, and heavy metals. Abiotic stresses have negative impact on the physiology and morphology of plants through defects in the genetic regulation of cellular pathways. Plants employ several tolerance mechanisms and pathways to avert the effects of stresses that are triggered whenever alterations in metabolism are encountered. Phytohormones are among the most important growth regulators; they are known for having a prominent impact on plant metabolism, and additionally, they play a vital role in the stimulation of plant defense response mechanisms against stresses. Exogenous phytohormone supplementation has been adopted to improve growth and metabolism under stress conditions. Recent investigations have shown that phytohormones produced by root-associated microbes may prove to be important metabolic engineering targets for inducing host tolerance to abiotic stresses. Phytohormone biosynthetic pathways have been identified using several genetic and biochemical methods, and numerous reviews are currently available on this topic. Here, we review current knowledge on the function of phytohormones involved in the improvement of abiotic stress tolerance and defense response in plants exposed to different stressors. We focus on recent successes in identifying the roles of microbial phytohormones that induce stress tolerance, especially in crop plants. In doing so, this review highlights important plant morpho-physiological traits that can be exploited to identify the positive effects of phytohormones on stress tolerance. This review will therefore be helpful to plant physiologists and agricultural microbiologists in designing strategies and tools for the development of broad spectrum microbial inoculants supporting sustainable crop production under hostile environments.

## Introduction

The Food and Agricultural Organization has provided an estimate of the alarmingly increasing human population, expected to reach 8–9 billion by 2030 (FAO, 2010). As a result of increasing urbanization and industrialization, threats to the environment have increased, leading to the shrinkage of agricultural land on one hand and causing significant declines in crop growth on the other hand. Abiotic stresses have the potential to restrict the growth of crop plants considerably, therefore leading to significant yield losses and posing a potential threat to global food security (Mahalingam, 2015).

Environmental stresses are detrimental to the growth of plants. Drought, salinity, heavy metal contamination, flooding, temperature (cold and high), and ultraviolet radiation are the key abiotic factors that modulate the growth of plants to the extent that a reduction in yield is a certain effect. Changes in the climate patterns of different regions have resulted in shifts in vegetation, and approximately 2,000 million hectares of land worldwide has been affected by increased water scarcity and salinization (El-Beltagy and Madkour, 2012). It is believed that approximately 25% of global agricultural land is affected by drought and approximately 5–7% is affected by salt (Ruiz-Lozano et al., 2012). Abiotic stresses inhibit plant growth by reducing water uptake and altering plant physiological and biochemical processes (Ahmad et al., 2010; Hashem et al., 2016). Heavy metals, including cadmium, lead, and mercury, are toxic and are mostly present in soils at low concentrations. However, due to their high mobility in the soil–plant system, they are readily taken up by plants and delivered to the shoot (Hart et al., 1998). Increases in metal concentrations cause retardation of growth, leading to necrosis, altered nutrient uptake, reduced enzyme activity and hence phytotoxicity (Groppa et al., 2012).

A better understanding of the different tolerance strategies for maintaining crop productivity through the manipulation of environmental conditions can be helpful for maintaining the maximum genetic potential of crops as much as possible. Phytohormones are important growth regulators synthesized in defined organs of the plant that have a prominent impact on plant metabolism (Kazan, 2013) and play an important role in the mitigation of abiotic stresses (Teale et al., 2006; Hu et al., 2013). However, abiotic stresses alter the endogenous levels of phytohormones, such as auxins, gibberellins, abscisic acid (ABA), jasmonic acid and salicylic acid (SA), which causes plant growth perturbations (Debez et al., 2001; Egamberdieva, 2009; Khan et al., 2014). Drought and salt stress have also been reported to inhibit phytohormone concentrations in plant tissue.

There has been enormous progress in research regarding crop improvement in hostile environments, and the role of some tools, such as microbial technology and genetic engineering, has been acknowledged. Accordingly, several strategies for improving plant stress tolerance by root-associated microbes, such as a low-input biotechnology, have been proposed (Khan et al., 2013). Plant-associated microbes live in plant tissue endophytically or symbiotically or they colonize the root surface and cooperate with each other by producing various metabolically active substances (Egamberdieva, 2011, 2012; Berg et al., 2013; Asaf et al., 2017). The stimulation of plant growth and nutrient acquisition by beneficial rhizobacteria has been correlated to the biosynthesis of plant growth regulators, including auxins (Etesami et al., 2015; Pereira et al., 2016), gibberellins (Khan et al., 2014), cytokinins (Kudoyarova et al., 2014), and ABA (Sgroy et al., 2009). The microbial regulators modulate plant hormone levels in plant tissue, and they have been found to have effects that are similar to exogenous phytohormone application (Egamberdieva, 2009; Turan et al., 2014; Shahzad et al., 2016). Based on the currently available studies on the effect of phytohormones on plant stress tolerance, this review attempts to improve the understanding of microbial phytohormones and their interactions with plants by assessing their influence on plant physiological and morphological properties. Based on important studies on the negative effect of abiotic stresses on plant growth regulators, we have also presented some potential traits of microbial phytohormones that can be used to increase plant growth and tolerance to stress factors. In this review, we will focus on the plant growth regulators synthesized by root-associated microbes, their diversity, physiology and their involvement in stress tolerance of plants to abiotic stresses including drought, salt, and heavy metals.

## Role of Phytohormones in Plant Response to Abiotic Stress

### Auxins

Auxins are important phytohormones, and the auxin indole-3-acetic acid (IAA) was shown to promote several growth and developmental events, such as cell division, elongation, and differentiation (Asgher et al., 2015). IAA is synthesized from and chemically similar to tryptophan. Ljung (2013) produced strong evidence favoring auxin-mediated growth and developmental control through alterations in gene expression patterns. Many reports are available depicting varied modulations in the synthesis, transport, metabolism and activity of auxins after plant exposure to stresses (Ljung, 2013); however, plenty of research reports are available advocating the role of auxins in mediating and improving plant tolerance to abiotic stresses (Kazan, 2013). Rice plants exhibited a significant decline in IAA after exposure to salinity stress. In addition, this variation in IAA can induce growth modulation through an increase in other phytohormones, such as ABA, as reported by Iqbal and Ashraf (2013). Jung and Park (2011) found a link among auxin signaling and salt stress which developed through auxin involvement in modulating the membrane bound transcription factor NTM2. These involvements were further validated by over-expression studies on the IAA30 gene of NTM2 carried out by Park et al. (2011); however, the actual mechanism of IAA-induced mitigation of salinity is unclear.

Auxins have an important role, whether directly or indirectly, in promoting heavy metal tolerance, as Hu et al. (2013) observed that heavy metals have a negative effect on the biosynthesis of auxins. The toxic effect of lead (Pb) on sunflower plant growth was alleviated by the addition of a low concentration of IAA (10^-10^ M), which stimulated increases in root volume, surface area and diameter (Fässler et al., 2010). IAA induced an increase in shoot biomass and increased Pb and Zn accumulation in plant tissue, indicating the potential of auxins to enhance the phytoextraction of metals. Aluminum restricts root growth in *Medicago sativa* by reducing the transport and synthesis of IAA from shoot to root, which was confirmed after analyzing the expression of genes; however, exogenous application of IAA was observed to mitigate aluminum stress to some extent by maintaining greater expression of the AUX1 and PIN2 genes (Wang S. et al., 2016). There was a positive effect after using auxins as priming sources. Iqbal and Ashraf (2007) have reported a significant mitigation of salt stress-induced hostile effects in wheat after seed priming with IAA, which resulted in ionic homeostasis and induction of SA biosynthesis. These studies indicate the existence of possible crosstalk between auxin and SA that mediates tolerance responses in plants. Salinity restricts the synthesis of IAA; however, the exogenous application of SA proved effective in mitigating hostile effects by causing significant alleviation of salinity-induced inhibition (Fahad and Bano, 2012).

### Cytokinins

Cytokinins (CK), an important group of plant hormones are involved in the maintaining of cellular proliferation and differentiation and the prevention of senescence, therefore leading to the inhibition of premature leaf senescence (Schmulling, 2002). However, under stress conditions, particularly water stress at the grain-filling stage, it was observed that stay-green genotypes have the potential to exhibit increased tolerance, which was ascribed to an increased concentration of cytokinin in the xylem sap (Borrell et al., 2000). Zhang et al. (2010) demonstrated that cytokinin-over-expressing transgenic cassava exhibited greater tolerance to drought in comparison to wild-type plants. The genes involved in the biosynthesis of cytokinin have been over-expressed, and their role in stress tolerance has been validated. For example, the ipt gene has been validated in field analysis (Peleg and Blumwald, 2011). Reduced cytokinin leads to ABA-induced stomatal closure, thereby reducing carbon uptake and assimilation, and under stressful conditions, the up-regulation of cytokinin oxidase may also reduce carbon metabolism; work on this topic can be fruitful in improving the plant growth and yield. Mohapatra et al. (2011) demonstrated that cytokinin improves grain filling. Currently, exogenous application of cytokinin is being employed to optimize the internal concentrations of cytokinin. It has also been documented that heavy metals, such as zinc and lead, severely hamper the seedling growth of chickpea through the inhibition of GA_3_ and Z concentrations in plant tissue (Atici et al., 2005). In an earlier report, the application of kinetin to chickpea stimulated plant growth and development under salt stress (Bozcuk, 1981), and in another report, kinetin alleviated cadmium stress in eggplant by enhancing its antioxidant potential (Singh and Prasad, 2014).

### Abscisic Acid

Like other phytohormones, ABA is known to have an important role in plants by improving stress responses and adaptation. It is a naturally occurring sesquiterpenoid, which are a group of key phytohormones involved in the regulation of growth. There have been many reports advocating the role of ABA in integrating signaling during stress exposure with subsequent control of downstream responses (Wilkinson et al., 2012). Under abiotic stress the expression of stress responsive genes regulated by ABA-induced and -mediated signaling, leading to better elicitation of tolerance responses (Sah et al., 2016). In addition, ABA has been reported to control root growth and water content under drought stress conditions (Cutler et al., 2010). However, an abrupt increase in ABA concentrations during stress exposures can lead to growth retardation and can also modulate tolerance responses against stresses (Asgher et al., 2015). Nevertheless, there are reports suggesting the positive implication of exogenous ABA in reversing the ill effects of stresses, such as salinity (Gomez et al., 2002), chilling (Nayyar et al., 2005), drought (Bano et al., 2012), and cold stress (Li et al., 2014). Bano et al. (2012) demonstrated that exogenous application of ABA protected wheat from drought-induced oxidative damage by improving the antioxidant system and relative water content. Exogenous ABA application for improving stress tolerance has been proposed as an effective tool for stress mitigation. In *Solanum tuberosum*, Mora-Herrera and Lopez-Delgado (2007) observed that ABA application resulted in improved stress tolerance by reducing the production of free radicals through significant increases in the activity of the antioxidant enzyme peroxidase. Zhou et al. (2014) observed a significant alteration in the proteome of tea due to exogenous application of ABA under drought stress conditions, including changes in proteins involved in transport, carbon metabolism, and stress tolerance. It has been suggested that ABA maintains the levels of other hormones, such as ethylene, leading to the maintenance of shoot and root growth in *Zea mays* (Spollen et al., 2000). Upon stresses, ABA synthesis and accumulation in plant tissue increases. The most important role of ABA, in addition to its role in signaling, is its ability to act as an anti-transpirant after the induction of stomatal closure and reduction of canopy expansion (Wilkinson and Davies, 2002). Exogenous ABA application to rice seedlings exposed to drought led to the protection of photosynthesis by up-regulating the expression of the OsPsbD1, OsPsbD2, OsNCED2, OsNCED3, OsNCED4 and OsNCED5 genes, leading to improved photosynthetic capacity, and stomatal regulation under normal and stressed conditions, which suggests the involvement of these genes in photosystem II induction after exogenous ABA application. In plants exposed to stress conditions, ABA is involved in developing the deeper root system and causing other necessary root modifications to mediate optimal water and nutrient acquisition (Spollen et al., 2000; Vysotskaya et al., 2009). In addition, ABA maintains the hydraulic conductivities of shoot and root to better exploit soil water content, leading to the maintenance of tissue turgor potential and improved drought tolerance through up-regulation of the antioxidant system and the accumulation of compatible osmolytes (Chaves et al., 2003), which maintains the relative water content. In *Stylosanthes guianensis*, Zhou et al. (2005) demonstrated that ABA-induced antioxidant defense was mediated by improved nitric oxide synthesis. Guajardo et al. (2016) also reported improved activity of antioxidant enzymes after ABA treatment, which resulted in increased desiccation tolerance by reducing oxidative stress-induced damage. Cabot et al. (2009) reported that ABA applied exogenousely inhibited accumulation of sodium and chloride in citrus plants with exogenously applied ABA. In another study, ABA treatment increased plant growth, nutrient uptake, and nitrogen fixation in the common bean under salt stress (Khadri et al., 2006).

### Gibberellic Acid

Another important plant growth regulator is gibberellin, which has a vital role in seed dormancy formation of floral organs, and lateral shoot growth (Olszewski et al., 2002). The available literature clearly reveals the ameliorative impact of gibberellic acid against salinity. Gibberellic acid was found to stimulate plant growth and development under various abiotic stress conditions (Ahmad, 2010). Enhanced plant water uptake and reduced stomatal resistance were observed in gibberellic acid-treated tomato plants grown under saline conditions (Maggio et al., 2010). Gibberellic acid induces efficient uptake and ion partitioning within the plant system, leading to enhanced growth and maintaining the metabolism of plants under normal and stress conditions (Iqbal and Ashraf, 2013). Under salt stress conditions, improved germination and growth due to gibberellic acid has been reported by several studies (Tuna et al., 2008; Ahmad, 2010; Manjili et al., 2012). In addition, gibberellins can exhibit crosstalk with other phytohormones, which elicits important responses and mediates tolerance mechanisms for enhancing stress tolerance. The synthesis of gibberellins can also be promoted through the application of other hormones, such as auxin (Wolbang et al., 2004). Enhanced synthesis of gibberellic acid leads to enhanced ABA catabolism. Moreover, gibberellic acid directly affects growth, yield, and mineral nutrition as well as nitrogen metabolism. Khan et al. (2004) reported increases in fruit yield, leaf area, and nitrogen, phosphorous, and potassium uptake in tomato due to the exogenous application of gibberellic acid. Moreover, an increase in osmotic components was reported in plants exposed to salt stress, and their content was further increased by gibberellic acid treatment. The endogenous application of GA resulted in amendment of osmotic stress in plants and maintenance of tissue water content (Ahmad, 2010). Such effects were observed by Manjili et al. (2012) for wheat and by Tuna et al. (2008) for maize. In addition, gibberellic acid enhanced antioxidant enzyme activity by lowering the levels of reactive oxygen species (ROS) which contributed to better growth under stress (Manjili et al., 2012). In addition, exogenous application of gibberellic acid mitigates salinity-induced effects on germination and growth in *Arabidopsis thaliana* by mediating enhanced synthesis of SA, which causes increased activity of isochorismate synthase 1 (Alonso-Ramirez et al., 2009). The same study also demonstrated that over-expression of the gibberellin-responsive gene from *Fagus sylvatica* enhanced the salt tolerance of *Arabidopsis*.

### Salicylic Acid

Salicylic acid is another important phytohormone with a phenolic nature, and it has an important function in plant stress tolerance through modulation of antioxidative enzyme activities (Ahmad et al., 2011; da Silva et al., 2017). The alleviation of various abiotic stresses by application of SA was reported by Senaratna et al. (2000) for water stress, by Azooz et al. (2011) for salt stress and by Ahmad et al. (2011) for heavy metal stress. SA modulates several physiological processes involved in plant stress tolerance through stress activated signal pathways and response mechanisms (Ahmad et al., 2011; Janda et al., 2012; Khan et al., 2014). There are several reports on the alleviating effect of SA in plants, e.g., fava bean (Azooz et al., 2011), maize (Gunes et al., 2007), and wheat (Shakirova, 2007). Azooz et al. (2011) reported that the application of SA to sea water-treated *Vicia faba* plants not only ameliorated the negative effects on growth, biomass accumulation and antioxidant system but also caused efficient accumulation of organic osmolytes, such as proline and free amino acids. In salt-stressed *Vigna radiata*, L. Khan et al. (2014) reported a reduction in endogenous levels of ethylene due to SA application. The results published so far have shown that the application of SA promoted efficient sequestration and partitioning of deleterious ions, such as Na. Increased synthesis and accumulation of proline and ABA have been reported in salinity-stressed wheat seedlings, contributing to better growth and yield (Shakirova et al., 2003). In *Vigna radiata*, L. Khan et al. (2014) observed that treatment of seeds with SA helped to considerably mitigate salt stress-induced changes. SA-treated plants showed better growth in terms of biomass accumulation, promotion of cell division, and showed a higher photosynthetic rate and antioxidant enzyme activity (da Silva et al., 2017). In barley plants, salinity stress caused alterations in the rate of photosynthesis and membrane stability; however, these negative effects of salinity stress were ameliorated by the application of SA (Janda et al., 2012). Similar observations were reported by da Silva et al. (2017) in case of SA at 10^-5^ M which increased plant growth of sesame under drought stress (da Silva et al., 2017). The treatment of maize with SA reduced the accumulation of Na in plant tissue and mitigated salt-induced negative effects on plants (Gunes et al., 2007). In addition, SA inhibits lipid peroxidation, improves membrane stability (Azooz et al., 2011), sustains the transpiration rate, and decreases electrolyte leakage (Stevens et al., 2006). Tang et al. (2017) reported that SA application mitigates water stress by maintaining a lower ROS level. Several studies reported increased mitochondrial alternative oxidase (AOX) expression and activity by SA treatment; this enzyme plays an important role in tolerance to abiotic stresses (Zhang et al., 2010).

Altogether, these observations suggest that phytohormones play a vital role in plant tolerance to various abiotic stresses by modulating the physiological properties and defense system of plants. Since plants are closely associated with the microbes that live within plant tissues, microbial metabolites may have strong effect on plant physiological processes and metabolism. In earlier studies, Fulchieri et al. (1993) and Lucangeli and Bottini (1997) observed higher amounts of IAA and the gibberellin GA3 in the plant tissue of maize after the inoculation of plant growth promoting rhizobacteria (PGPR) strains. Similar observations were also reported by Fulchieri et al. (1993) in which *Azospirillum* increased levels of GA3 in maize seedlings. Thus, microbial phytohormones have vital importance in plant host metabolism and physiology under hostile environments.

## Root-Associated Phytohormone-Producing Microbes

Soils are sources of diverse organisms, including fungi, bacteria, and plants (Mendes et al., 2013). Plant roots are heavily colonized with microorganisms (compared to soil and other habitats) because of the rich nutrient component of root exudates (Schlaeppi and Bulgarelli, 2015; Hashem et al., 2016). The rhizosphere is a relatively nutrient-rich environment containing amino acids, sugars, fatty acids and other organic compounds, which attract microbes (Vorholt, 2012) that utilize the various nutrients released by the root. In turn, the microbes synthesize biologically active compounds, including phytohormones (auxins, cytokinins, gibberellins, and ABA), antifungal compounds, enzymes, and compatible solutes. These microbial metabolites play a vital role in plant growth, nutrition and development (Ruiz-Lozano et al., 2012; Sorty et al., 2016; Egamberdieva et al., 2017a). They can stimulate plant growth development, provide resistance to various abiotic and biotic stress factors, improve nutrient acquisition and protect plants from various soil-borne pathogens (Grover et al., 2013; Cho et al., 2015). The beneficial interactions of microbes in plants, their positive effect on plant growth and their improvement of stress tolerance under extreme environmental conditions have been extensively reviewed by Nadeem et al. (2014), and the mechanisms utilized by plant growth-promoting bacteria have been reviewed by Forni et al. (2017). There are several mechanisms of plant growth stimulation, plant protection and alleviation of salt stress by PGPR, such as nitrogen fixation; synthesis of osmoprotectants, exopolysaccharides, 1-aminocyclopropane-1-carboxylate (ACC) deaminase, cell wall degrading enzymes, and phytohormones; modulation of antioxidant enzymes or nutrients; and solubilization of minerals, such as phosphorus, and potassium (Berg et al., 2013; Wang Q. et al., 2016; Mishra et al., 2017). The microbes mitigate stress responses by regulating the nutritional and hormonal balance in plants and inducing systemic tolerance to stress. One of the mechanisms of improvement of plant growth and stress tolerance by microbes is their phytohormone synthesizing ability in the rhizosphere or root tissue (Etesami et al., 2015). Microbial phytohormones affect the metabolism of endogenous growth regulators in plant tissue (Hashem et al., 2016; Sorty et al., 2016) and play a key role in changing root morphology upon exposure to drought, salinity, extreme temperature and heavy metal toxicity (Spaepen et al., 2008; Khan et al., 2011).

Root-associated microbes, including free living, symbiotic or endophytic microbes, can produce various type of phytohormones and belong to different genera and species (Sgroy et al., 2009). For example, Sorty et al. (2016) isolated diverse groups of organisms belonging to *Acinetobacter, Bacillus, Enterobacter, Pantoea, Pseudomonas, Rhizobium*, and *Sinorhizobium* from halotolerant weed (*Psoralea corylifolia* L.), and Egamberdieva et al. (2016) found *Arthrobacter, Bacillus, Enterobacter, Pseudomonas, Rhizobium, Brevibacillus, Cellulosimicrobium, Mycobacterium, Ochrobactrum, Paenibacillus*, and *Pseudoxanthomonas* associated with soybean root. The IAA-producing *Mycobacterium* species was observed in the rhizosphere of orchid (Tsavkelova et al., 2007), and *Azotobacter, Azospirillum, Cellulomonas, Mycoplana*, and *Rahnella* were found in the wheat rhizosphere (Egamberdiyeva and Hoflich, 2003; Egamberdieva et al., 2008). In other reports, *Pseudomonas* spp. (Lawongsa et al., 2008), *Arthrobacter* spp. (Piccoli et al., 2011), and *Enterobacter, Pseudomonas*, and *Stenotrophomonas* species were associated with plants that produced IAA (Khan and Doty, 2009). Piccoli et al. (2011) isolated the endophytic diazotrophic bacterium *Arthrobacter koreensis* which produce ABA, IAA, GA3 and jasmonic acid from the roots of the halophyte shrub *Prosopis strombulifera*. The endophytic strains of *Klebsiella* and *Enterobacter* isolates from sugar cane synthesize IAA (de Santi Ferrara et al., 2012). Mishra et al. (2017) isolated bacteria with IAA production ability from extreme environments, which were identified as *Pseudomonas* spp. and *Ochrobactrum* spp. In other studies *Halomonas desiderata, Bacillus megaterium, Bacillus cereus, Bacillus subtilis, Escherichia coli*, and *Pseudomonas fluorescens* G20-18 were reported to synthesize cytokinins (Salamone et al., 2001; Karadeniz et al., 2006; Großkinsky et al., 2016). Bacterial isolates from the rhizosphere of a vegetable (bitter gourd) belonging to genera *Bacillus, Klebsiella, Leifsonia*, and *Enterobacter* were able to produce IAA and improved maize growth in Cd-contaminated soil (Ahmad et al., 2016).

Naz et al. (2009) also observed cytokinin-producing species, such as *Arthrobacter, Bacillus, Azospirillum*, and *Pseudomonas*, that stimulated the root development of plants. ABA was also detected in root-associated microbes from various plants. Karadeniz et al. (2006) reported *Proteus mirabilis, Phaseolus vulgaris, Klebsiella pneumoniae, B. megaterium*, and *B. cereus* as ABA-producing bacteria. Species such as *Bacillus pumilus, Bacillus licheniformis, Acetobacter* sp., *Bacillus* sp., *Azospirillium* sp. were found among gibberellin-producing strains (Gutiérrez-Mañero et al., 2001; Bottini et al., 2004). Salomon et al. (2014) observed ABA-producing *B. licheniformis* Rt4M10 and *P. fluorescens* Rt6M10 in the rhizosphere of *Vitis vinifera*. *Achromobacter xylosoxidans* SF2, isolated from sunflower roots, was also able to produce ABA in minimal medium (Forchetti et al., 2007). Among IAA-producing bacteria associated with plants grown under saline soil, Rhizobia have also been shown to synthesize auxins, cytokinins and abscicic acids, increase plant growth and development and improve the yield of agricultural crops (Hayat et al., 2008). Actinobacteria have also been found to produce IAA, CK, GB-like substances (Shutsrirung et al., 2013; Vijayabharathi et al., 2016). Ruanpanun et al. (2010) found high IAA-producing nematophagous actinomycete and fungal isolates, such as *Aspergillus* and S*treptomyces*. In other studies, *Streptomyces* sp. Isolated from medicinal plant species *Taxus chinensis* and *Artemisia annua* showed IAA synthesis ability (Lin and Xu, 2013). Shutsrirung et al., 2013 reported IAA production in endophytic actinomycetes *Streptomyces, Nocardia, Nocardiopsis, Spirillospora, Microbispora*, and *Micromonospora* associated with mandarin.

The stress tolerance ability of bacterial strains provides important benefits to plants. The ability of root-associated microbes to synthesize phytohormones is typically not hampered by high salt concentrations (Egamberdieva and Kucharova, 2009). For example, phytohormone synthesis by endophytic actinobacteria *Streptomyces coelicolor* DE07 and *Streptomyces geysiriensis* DE27 was not inhibited under water stress (Yandigeri et al., 2012). The production of IAA by *A. brasilense* in osmotic stress conditions was higher than that of osmosensitive *A. brasilense* Sp7 (Nabti et al., 2007). In another study, *Pseudomonas putida, Pseudomonas extremorientalis, Pseudomonas chlororaphis*, and *P. aurantiaca* were able to produce IAA in a 4% NaCl conditions (Egamberdieva and Kucharova, 2009). *Pseudomonas* sp. and *Bacillus* sp. strains were able to produce IAA under high salt conditions (200–400 mM NaCl) and increased the plant biomass of *Sulla carnosa* under salt stress (Hidri et al., 2016).

The biosynthesis of phytohormones differs by bacterial strain. For example, *Bacillus* and *Pseudomonas* strains synthesized IAA concentrations up to 2.2 μg mL^-1^, GA3 production by *A. xylosoxidans* and *B. halotolerans* was between 36.5 and 75.5 μg mL^-1^ (Sgroy et al., 2009). In addition, ABA production was 0.3, 1.8, and 4.2 μg mL^-1^ in the culture medium of *L. fusiformis* (Ps14), *B. subtilis* (Ps8), and *P. putida* (Ps30), respectively. In another study, *Bacillus amyloliquefaciens* associated with rice (*Oryza sativa* L.) synthesized gibberellins, and the quantities of GA differed, e.g., 17.8 ng mL^-1^ for GA20, 5.7 ng mL^-1^ for GA36, 5.6 ng mL^-1^ for GA24, 1.02 ng mL^-1^ for GA4, 0.7 ng mL^-1^ for GA53, 0.08 ng mL^-1^ for GA5, and 0.01 ng mL^-1^ for GA8 (Shahzad et al., 2016). Endophytic fungi *Aspergillus fumigatus* associated with soybean roots synthesized gibberellins, such as GA4 (24.8 ng mL^-1^), GA9 (1.2 ng mL^-1^), and GA12 (9.8 ng mL^-1^) (Khan et al., 2011). Several studies reported SA production by root-associated bacteria, e.g., *B. licheniformis* MML2501 (18 μg mL^-1^) (Shanmugam and Narayanasamy, 2008), and *Pseudomonas* sp. PRGB06 (6.8 μg mL^-1^) (Indiragandhi et al., 2008).

## Microbial Phytohormones in Plant Stress Tolerance

**FIGURE 1:**
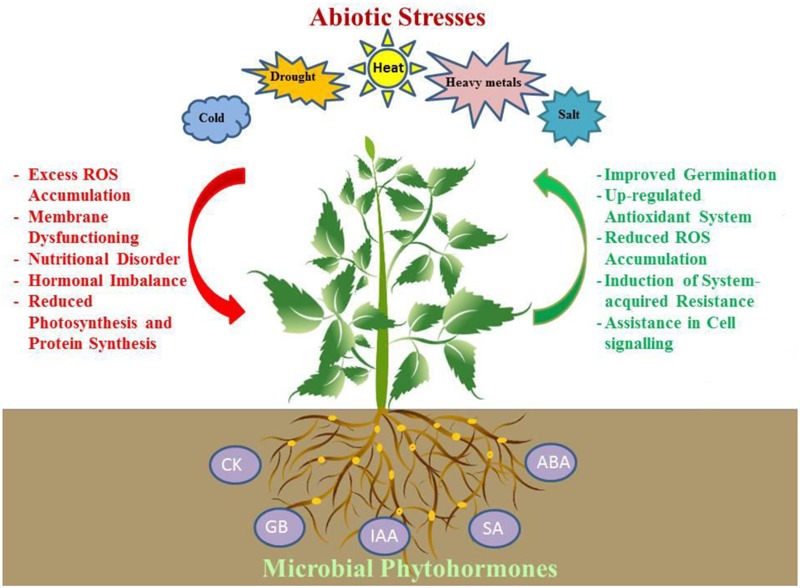
An overview of mechanisms in microbial phytohormone-mediated plant stress tolerance. Several root associated microbes produce cytokinin (CK), gibberellin (GB), indole-3-acetic acid (IAA), salicylic acid (SA) and abscisic acid (ABA), which help plants to withstand stress by enhancing its antioxidant potential, by up-regulation of the antioxidant system and by accumulation of compatible osmolytes thus reducing oxidative stress-induced damage; improving photosynthetic capacity and membrane stability; promoting cell division and stomatal regulation; stimulating growth of root system, and acquisition of water and nutrients.

Microbes synthesize low amounts of phytohormones and improve stress tolerance and plant growth under various stress conditions, including salinity, heat, drought and metal toxicity, as reported in many studies (Sgroy et al., 2009; Egamberdieva et al., 2011, 2017b; Liu Y. et al., 2013). The beneficial effect of phytohormone-producing microbes on alleviating abiotic stress in plants was reported in numerous studies (**Figure 1**; Khan and Doty, 2009; Ngumbi and Kloepper, 2014; Hashem et al., 2016). Some examples of phytohormone-producing bacteria and their ability to mitigate abiotic stress are given in **Table 1**. Many studies have reported the positive effects of bacteria associated with plants and IAA production on plant growth stimulation under abiotic stress conditions. For example, bacterial strains *Curtobacterium flaccumfaciens* E108 and *Ensifer garamanticus* E110 isolated from *Hordeum secalinum* stimulated plant biomass and salt stress resistance in barley (Cardinale et al., 2015). The root-colonizing halotolerant bacterium *B. licheniformis* HSW-16 was able to mitigate salt stress-induced damage and stimulate the growth of wheat through the production of IAA under saline soil conditions (Singh and Jha, 2016). Similar observations were reported by Upadhyay et al. (2012) in which salt-tolerant bacterial strains *B. subtilis* and *Arthrobacter* sp. increased wheat biomass and total soluble sugars and reduced sodium concentration in plant tissue. Sorty et al. (2016) isolated salt-tolerant strain *Enterobacter* sp. NIASMVII from halotolerant weed (*Psoralea corylifolia* L.), which produces IAA (0.22 and 25.58 μg mL^-1^) and enhances seed germination of wheat (*Triticum aestivum* L.). In another study, *Pseudomonas* spp. isolated from extreme environments (close to the sites of volcanos) synthesized IAA under salt stress (500 mM NaCl) and high temperature (40°C), and they were able to stimulate increases in the root and shoot biomass of maize (Mishra et al., 2017). According to Bianco and Defez (2009), protection of plants from negative effects of abiotic stress by IAA is related to enhanced cellular defense systems. Several salt-tolerant strains synthesizing IAA in culture medium, namely, *Serratia plymuthica* RR-2-5-10, *Stenotrophomonas rhizophila* e-p10, *P. fluorescens* SPB2145, *P. extremorientalis* TSAU20, and *P. fluorescens* PCL1751, improved cucumber biomass and yield in greenhouse conditions (9–24%) (Egamberdieva et al., 2011). Root-associated IAA-producing bacteria were found to improve drought stress in plants. Marulanda et al. (2009) observed increased plant biomass in clover (*Trifolium repens* L.) after seed treatment with *P. putida* and *B. megaterium* under drought, and they found a correlation between these changes and increased IAA. IAA-producing bacteria were also found to improve plant growth and development under nutrient-poor soil conditions. *Serratia* sp. isolated from chickpea nodules was found to produce IAA, which led to an increased grain yield of chickpea in nutrient-deficient soil (Zaheer et al., 2016). Many fungal species were also able to produce plant growth regulators and alter plant root system and physiology. Contreras-Cornejo et al. (2009) observed increased lateral root formation, root hair growth and modified root system architecture from *Trichoderma virens* inoculation, which resulted in increased plant biomass of *Arabidopsis thaliana*.

**Table 1:** Some examples of phytohormone-producing bacteria and their ability to mitigate abiotic stress.

Microorganisms	Phytohormone	Host plant, abiotic stress	Reference
*Pseudomonas* sp., *Bacillus* sp.	IAA	*Sulla carnosa* (Desf.), Salt stress	Hidri et al., 2016
*Bacillus licheniformis*	IAA	*Triticum aestivum* L., Salt stress	Singh and Jha, 2016
*Bacillus subtilis, Arthrobacter* sp.	IAA	*Triticum aestivum* L., Salt stress	Upadhyay et al., 2012
*Pseudomonas putida, Bacillus megaterium*	IAA	*Trifolium repens*, Drought stress	Marulanda et al., 2009
*Marinobacterium* sp., *Pseudomonas* sp., *Rhizobium* sp., *Sinorhizobium* sp.	IAA	*Triticum aestivum* L., Salt stress	Sorty et al., 2016
*Serratia plymuthica, Stenotrophomonas rhizophila, Pseudomonas fluorescens, Pseudomonas extremorientalis*	IAA	*Cucumis sativus*, Salt stress	Egamberdieva et al., 2011
*Acinetobacter faecalis, Bacillus cereus, Enterobacter hormaechei, Pantoea agglomerans*	IAA	*Triticum aestivum* L., Salt stress	Egamberdieva et al., 2008
*Curtobacterium flaccumfaciens, Ensifer garamanticus*	IAA	*Hordeum vulgare*, Salt stress	Cardinale et al., 2015
*Streptomyces coelicolor, Streptomyces geysiriensis*	IAA	*Triticum aestivum* L., Salt stress	Yandigeri et al., 2012
*Bacillus subtilis*	IAA	*Acacia gerrardii* Benth., Salt stress	Hashem et al., 2016
*Pseudomonas* sp.	IAA	*Zea mays*, Salt and heat stresses	Mishra et al., 2017
*Serratia* sp.	IAA	*Cicer arietinum* L., Nutrient stress	Zaheer et al., 2016
*Achromobacter xylosoxidans*	IAA	*Brassica juncea*, Cu stress	Ma et al., 2008
*Pseudomonas putida*	IAA	*Glycine max* (L.) Merr., Salt stress	Egamberdieva et al., 2017b
*Leifsonia* sp., *Bacillus* sp.	IAA	*Zea mays*, Cd stress	Ahmad et al., 2016
*Burkholderia* sp.	IAA	*Solanum lycopersicum* L., Cd stress	Dourado et al., 2013
*Bacillus subtilis*	IAA	*Brassica juncea* L., Ni stress	Zaidi et al., 2006
*Bacillus megaterium*	IAA	*Vinca rosea* L., Ni stress	Khan et al., 2017
*Achromobacter xylosoxidans, Bacillus pumilus*	SA	*Helianthus annuus*, Drought stress	Forchetti et al., 2010
*Serratia marcescens*	SA	*Zea mays*, Salt stress	Lavania and Nautiyal, 2013
*Micrococcus luteus*	CK	*Zea mays*, Drought stress	Raza and Faisal, 2013
*Arthrobacter* sp., *Bacillus* sp., *Azospirillum* sp.	CK	*Glycine max* (L.) Merr., Salt stress	Naz et al., 2009
*Bacillus subtilis*	CK	*Platycladus orientalis*, Drought stress	Liu F. et al., 2013
*Aspergillus fumigatus*	GA	*Glycine max* (L.) Merr., Salt stress	Khan et al., 2011
*Azospirillum lipoferum*	GA	*Triticum aestivum* L., Drought stress	Creus et al., 2004
*Phoma glomerata, Penicillium* sp.	GA	*Cucumis sativus*, Drought stress	Waqas et al., 2012
*Bacillus amyloliquefaciens*	ABA	*Oryza sativa* L., Salt stress	Shahzad et al., 2017
*Bacillus licheniformis, Pseudomonas fluorescens*	ABA	*Vitis vinifera* L., Water stress	Salomon et al., 2014
*Trichoderma asperellum*	IAA, GA, ABA	*Cucumis sativus*, Salt stress	Zhao and Zhang, 2015
*Bacillus aryabhattai*	IAA, GA, ABA	*Glycine max* (L.) Merr., Heat stress	Park et al., 2017

Microbial phytohormones also play an important role in metal-plant interactions, improving phytoextraction by plants. *A. xylosoxidans* Ax10 improved the root system of the *Brassica juncea* plant through IAA production activities, which increased copper phytoextraction (Ma et al., 2008). Similar results were observed by Zaidi et al. (2006) in which *B. subtilis* synthesizing IAA stimulated root growth and Ni accumulation in the Indian mustard plant (*B. juncea* L.). *B. megaterium* MCR-8, which produced auxin at a concentration of 68.5 mg 25 mL^-1^, alleviated Ni stress in *Vinca rosea* and stimulated root and shoot growth. In addition, plant treatment with *B. megaterium* MCR-8 increased the accumulation of total phenols, flavonoids and defense-related enzymes, such superoxide dismutase (SOD), catalase (CAT), peroxidase (POD), and ascorbate peroxidase (APX), compared to uninoculated plants under Ni stress (Khan et al., 2017). In another study, Ahmad et al. (2016) observed inhibited seed germination and seedling growth of maize by Cd stress, whereas Cd-tolerant and IAA-producing bacteria *Leifsonia* sp. and *Bacillus* sp. significantly increased shoot and root growth of maize in Cd-contaminated soil compared to controls. Similar observations were reported by Dourado et al. (2013) in which Cd-tolerant multi-tolerant bacteria *Burkholderia* sp. SCMS54 produced IAA and improved plant growth and stress tolerance of tomato to Cd stress. Islam et al. (2016) reported that Cr toxicity significantly inhibited maize growth, negatively affecting its physiological processes, such as photosynthetic pigment and carbohydrate metabolism, and increasing its levels of proline, H_2_O_2_, and MDA. In these conditions, Cr-resistant *P. mirabilis* isolates T2Cr and CrP450, combined with SA, mitigated the toxic effect of Cr, improved the root and shoot growth of maize and reduced oxidative stress in maize tissue by elevating its antioxidant activities.

**FIGURE 2:**
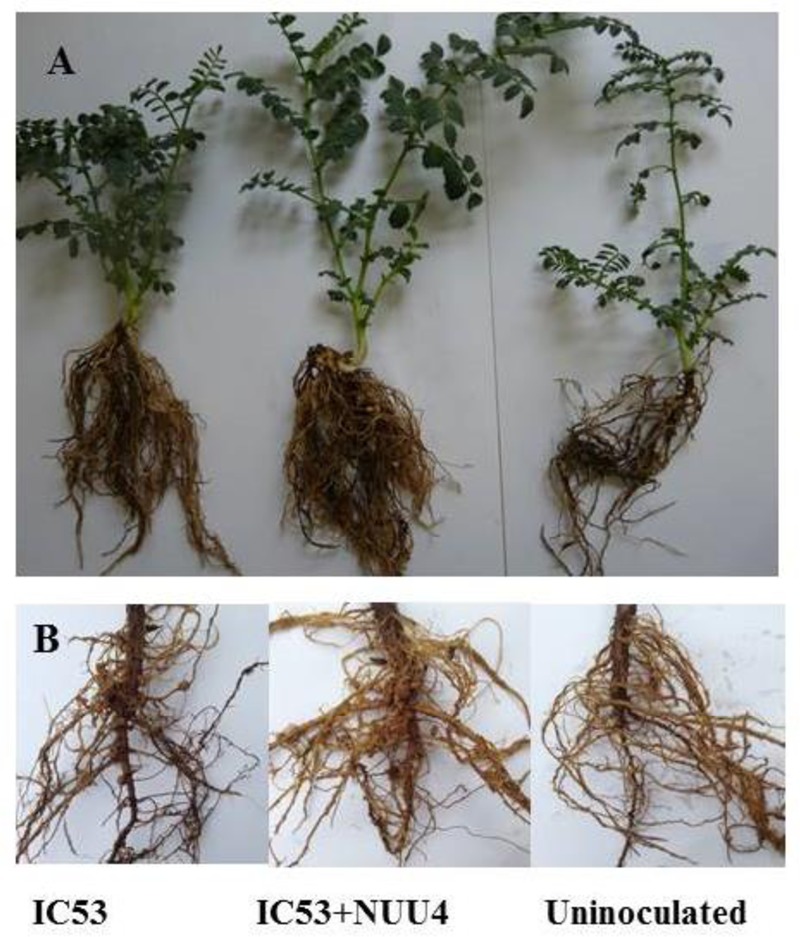
Growth of chickpea in salinated soil after inoculation with *Mesorhizobium ciceri* IC53 alone or with the combination of *Mesorhizobium ciceri* IC53 and IAA-producing *Bacillus subtilis* NUU4 in pots **(A)** and under field condition **(B)** (Figure as originally published in Egamberdieva et al., 2017c).

The tripartite interaction of root-associated microbes with symbiotic microbes and the host plant is also a mutualistic interaction that improves plant growth under stress through the induction of osmoregulation, hormonal balance, biochemical processes and changes in metabolic interfaces among partners (Nadeem et al., 2014; Park et al., 2017). IAA-producing *B. subtilis* NUU4 in combination with *Mesorhizobium ciceri* IC53 stimulated root and shoot biomass and improved nodule formation in *chickpea* (*Cicer arietinum* L.) under salt stress, as compared to uninoculated plants and plants inoculated with *Mesorhizobium ciceri* IC53 alone (Egamberdieva et al., 2017c) (**Figure 2**).

The positive effect on root development by cytokinin-producing bacterial strains was also reported in many studies. For example, inoculation of maize with cytokinin-producing bacteria *Micrococcus luteus* chp37 isolated from the desert of Pakistan stimulated shoot and root biomass by 54% and modulated the physiological properties of the plant, including photosynthetic pigments, under drought conditions (Raza and Faisal, 2013). The cytokinin-producing root-associated bacteria strains *Arthrobacter, Bacillus, Azospirillum*, and *Pseudomonas* increased soybean shoot and root biomass as well as proline content in plant tissue under salt stress (Naz et al., 2009). A similar observation was reported by Liu F. et al. (2013) in which cytokinin-producing *B. subtilis* stimulated root biomass of *Platycladus orientalis* (oriental thuja) by 13.9% and increased cytokinin concentration of 47.52% in leaves relative to respective controls under water stress conditions. The higher content of cytokinin in plant tissue contributed to stomatal opening and alleviated some of the detrimental effects of water stress.

*Aspergillus fumigatus* produced gibberellins, such as GA4 (24.8 ng mL^-1^), GA9 (1.2 ng mL^-1^), and GA12 (9.8 ng mL^-1^), which increased photosynthetic pigments, and shoot biomass of soybean under salt stress (Khan et al., 2011). *Azospirillum lipoferum*, which synthesizes GA, increased the stress tolerance of wheat to drought (Creus et al., 2004). Waqas et al. (2012) also reported improved salt and drought stress tolerance in cucumber plant by GA-producing endophytic fungi *Phoma glomerata* LWL2 LWL3, which produced GA1 (8.720 ng mL^-1^), GA3 (2.420 ng mL^-1^) and GA4 (0.220 ng mL^-1^), and *Penicillium* sp., which produced GA1 (5.33 ng mL^-1^) and GA3 (3.42 ng mL^-1^) in culture filtrate. The fungal inoculation resulted in increased root and shoot growth and nutrient uptake, and reduced stress by down-regulating ABA and modifying SA and jasmonic acid concentrations in plant tissue. It is known that ABA and SA act as defense signaling constituents (Shinozaki and Yamaguchi-Shinozaki, 2007).

Salomon et al. (2014) observed ABA production by *B. licheniformis* and *P. fluorescens* that stimulated plant growth of grapevine under water stress by inducing ABA synthesis. Shahzad et al. (2017) reported ABA production by *Bacillus amyloliquefaciens* RWL-1 (0.32 ± 0.015–0.14 ± 0.030 ng mL^-1^) under normal and saline conditions. Bacterial inoculation significantly increased root and shoot growth and the concentration of SA in plant tissue of rice under salt stress conditions. Park et al. (2017) isolated *Bacillus aryabhattai* strain SRB02 from the rhizosphere of soybean, and it significantly promoted the plant biomass and nodule number of soybean. The strains produced up to 2 ng mL^-1^ ABA in culture and increased the drought stress tolerance of soybean through stomatal closure under high temperatures (38°C) relative to control plants.

Similar to the effects of other phytohormones, SA-producing endophytic bacteria *A. xylosoxidans* and *B. pumilus* also enhanced the biomass of sunflower seedlings under drought conditions (Forchetti et al., 2010). Similar observations were reported by Lavania and Nautiyal (2013) in which salt-tolerant SA-producing *Serratia marcescens* NBRI1213 stimulated root and shoot growth as well as nutrient acquisition by maize, and furthermore increased plant stress tolerance to salinity.

Some bacteria may produce several types of phytohormones in plant tissue that interact to modulate important physiological processes in plants, including hormonal balance. *Sphingomonas* sp. LK11 and *Serratia marcescens* TP1 produced 12.31 and 10.5 μM mL^-1^ of IAA in the culture broths, which stimulated root and shoot growth of soybean through increases in ABA and gibberellin and a decrease in jasmonic acid content compared to levels in the control plants (Asaf et al., 2017). *Trichoderma asperellum* Q1, which produces IAA, GA and ABA, stimulated the biomass fresh weight of cucumber seedlings under salt stress in comparison to untreated control plants (Zhao and Zhang, 2015). In addition, the concentration of phytohormones IAA, GA and ABA in cucumber leaves were also increased after application of *Trichoderma asperellum* Q1 under salt stress. Similar observations were reported by Park et al. (2017) for soybean inoculated with *Bacillus aryabhattai* SRB02, which produces IAA, GA, and ABA. The root and shoot growth and heat stress of soybean plants increased after bacterial inoculation. In addition, higher concentrations of IAA, JA, GA12, GA4, and GA7 were observed in plant tissue of *Bacillus aryabhattai* SRB02-treated plants. Similar observations were reported for maize inoculated with ABA*-*producing *Azospirillum lipoferum* and *A*. *brasilense* sp. 245 in which bacterial treatment resulted in an increased concentration of ABA in plant tissue (Cohen et al., 2015). These studies demonstrate the involvement of phytohormone modulation in plant tissue by plant-associated microbes that induce the stress tolerance of plants.

## Conclusion and Future Prospects

Overall, evidence was provided that the exogenous application of phytohormones of microbial origin is an important tool for increasing the abiotic and biotic stress tolerance of plants, providing potential practical applications under changing or extreme environmental conditions. The beneficial effects on plants mediated by microbes, such as the stimulation of plant growth, tolerance to abiotic stresses and resistance to pathogens, are based on the microbes’ ability to produce auxins, gibberellins, SA, ABA, and cytokinins in plant tissues. Thus, plant-associated microbes hold the potential to modulate hormone levels and metabolism in plant tissue, especially in biochemical processes that can prevent the damaging effects of external stresses, such as drought, salinity, nutrient deficiency, or heavy metal contamination. Optimizing phytohormone balance in plant tissues under stress by beneficial microbes could be a crucial challenge in the development of sustainable approaches to crop production. More experimental studies on various plant species are needed to determine whether these are plant-specific traits and to better understand the mechanisms involved in the interactions between microbial metabolites and the host that help plants optimize their responses in hostile environments. More specifically, it can be worthwhile to employ loss-of-function or gain-of-function genetic mechanisms to explore the associated mechanisms or reveal the antagonistic or synergistic interactions of phytohormones. The identification of receptors leading to the expression of specific genes after the application of a microbial phytohormone is also an important topic. Furthermore, studies on the performance of phytohormone-producing microbes in field experiments are necessary, and they should include competition for nutrients and niches between the microbial inoculant and the indigenous microflora. Moreover, investigations of host-microbe-stress interactions and their involved mechanisms using omics-based approaches, such as proteomics, genomics, metagenomics, and metabolomics, are needed.

## Author Contributions

DE, SW, and EA designed and wrote the manuscript. AH and AA edited and helped in finalizing the manuscript.

## Conflict of Interest Statement

The authors declare that the research was conducted in the absence of any commercial or financial relationships that could be construed as a potential conflict of interest.
